# Membrane nanotubes facilitate the propagation of inflammatory injury in the heart upon overactivation of the β-adrenergic receptor

**DOI:** 10.1038/s41419-020-03157-7

**Published:** 2020-11-07

**Authors:** Jing Shen, Ji-Min Wu, Guo-Min Hu, Ming-Zhe Li, Wen-Wen Cong, Ye-Nan Feng, Shuai-Xing Wang, Zi-Jian Li, Ming Xu, Er-Dan Dong, You-Yi Zhang, Han Xiao

**Affiliations:** 1 Department of Cardiology and Institute of Vascular Medicine, Peking University Third Hospital, NHC Key Laboratory of Cardiovascular Molecular Biology and Regulatory Peptides, Key Laboratory of Molecular Cardiovascular Science, Ministry of Education, Beijing Key Laboratory of Cardiovascular Receptors Research, 100191 Beijing, China; 2 Key Laboratory of Xinjiang Endemic and Ethnic Diseases, Department of Physiology, School of Medicine Shihezi University, 832000 Shihezi, China

**Keywords:** Cell death, Mechanisms of disease

## Abstract

Acute sympathetic stress quickly induces cardiac inflammation and injury, suggesting that pathogenic signals rapidly spread among cardiac cells and that cell-to-cell communication may play an important role in the subsequent cardiac injury. However, the underlying mechanism of this response is unknown. Our previous study demonstrated that acute β-adrenergic receptor (β-AR) signaling activates inflammasomes in the heart, which triggers the inflammatory cascade. In the present study, β-AR overactivation induced inflammasome activation in both the cardiomyocytes and cardiac fibroblasts (CFs) of mice hearts following a subcutaneous injection of isoproterenol (ISO, 5 mg/kg body weight), a selective agonist of β-AR. In isolated cardiac cells, ISO treatment only activated the inflammasomes in the cardiomyocytes but not the CFs. These results demonstrated that inflammasome activation was propagated from cardiomyocytes to CFs in the mice hearts. Further investigation revealed that the inflammasomes were activated in the cocultured CFs that connected with cardiomyocytes via membrane nanotubes (MNTs), a novel membrane structure that mediates distant intercellular connections and communication. Disruption of the MNTs with the microfilament polymerization inhibitor cytochalasin D (Cyto D) attenuated the inflammasome activation in the cocultured CFs. In addition, the MNT-mediated inflammasome activation in the CFs was blocked by deficiency of the inflammasome component NOD-like receptor protein 3 (NLRP3) in the cardiomyocytes, but not NLRP3 deficiency in the CFs. Moreover, ISO induced pyroptosis in the CFs cocultured with cardiomyocytes, and this process was inhibited by disruption of the MNTs with Cyto D or by the NLRP3 inhibitor MCC950 and the caspase-1 inhibitor Z-YVAD-FMK (FMK). Our study revealed that MNTs facilitate the rapid propagation of inflammasome activation among cardiac cells to promote pyroptosis in the early phase of β-adrenergic insult. Therefore, preventing inflammasome transfer is a potential therapeutic strategy to alleviate acute β-AR overactivation-induced cardiac injury.

## Introduction

The overactivation of the sympathetic nervous system promotes the pathogenesis and progression of many cardiovascular diseases^1,2^, in which inflammation is activated and is a crucial factor for cardiac injury^3^. During acute sympathetic stress, cardiac injury occurs soon after acute sympathetic insult^4^, implying that cell-to-cell communication contributes to the spread of pathogenic signals. However, the mechanism remains unclear.

Our group demonstrated that acute β-adrenergic receptor (β-AR) overactivation rapidly triggers NOD-like receptor protein 3 (NLRP3) inflammasome activation in cardiomyocytes, which increases the inflammatory cytokines and macrophage infiltration^5^. The NLRP3 inflammasome is a protein complex consisting of apoptosis-associated speck-like protein containing a CARD (ASC), NLRP3, and pro-caspase-1^6^. Activation of the inflammasome causes the cleavage of pro-caspase-1 into caspase-1 (p20), resulting in either the maturation of interleukin-1β and interleukin-18 or the induction of inflammatory programmed cell death, called pyroptosis^7,8^. Our preliminary study found that a β-AR agonist, isoproterenol (ISO), did not activate the inflammasome in isolated cardiac fibroblasts (CFs). Nonetheless, in mice hearts, the inflammasome was activated early in both cardiomyocytes and CFs in vivo. These results suggest that cell-to-cell communication contributes to inflammasome transfer, which subsequently induces propagation of inflammation. However, the mechanism underlying the inflammasome transfer between different cells has not been elucidated.

Cells communicate with each other via both indirect paracrine and direct pathways, such as gap junctions, adhesion junctions, and membrane nanotubes (MNTs)^9,10^. MNTs, also called tunneling nanotubes, contain cytoskeleton microfilaments as the core structure, and partially contain microtubules^11,12^. MNTs mediate direct cell-to-cell connections over long distances and facilitate the transfer of various molecules and organelles between cells. Our group demonstrated that MNTs exist in the heart and mediate the transport of mitochondria from myofibroblasts to distressed cardiomyocytes to prevent hypoxia/reoxygenation-induced apoptosis^13^. As MNTs are “highways” between cardiac cells, we aimed to clarify whether MNTs mediate the rapid transfer of inflammasomes from cardiomyocytes to CFs during β-AR overactivation, and whether this process contributes to cardiac inflammatory injury.

In the present study, we activated β-AR in mouse hearts and isolated cardiac cells to determine whether the activated inflammasomes could be transferred from cardiomyocytes to CFs via MNTs. Furthermore, the pyroptosis of CFs was detected to clarify the function of the transferred activated inflammasomes in the CFs.

## Results

### ISO activated inflammasomes in the CFs in vivo, but not isolated neonatal CFs

**Fig. 1:**
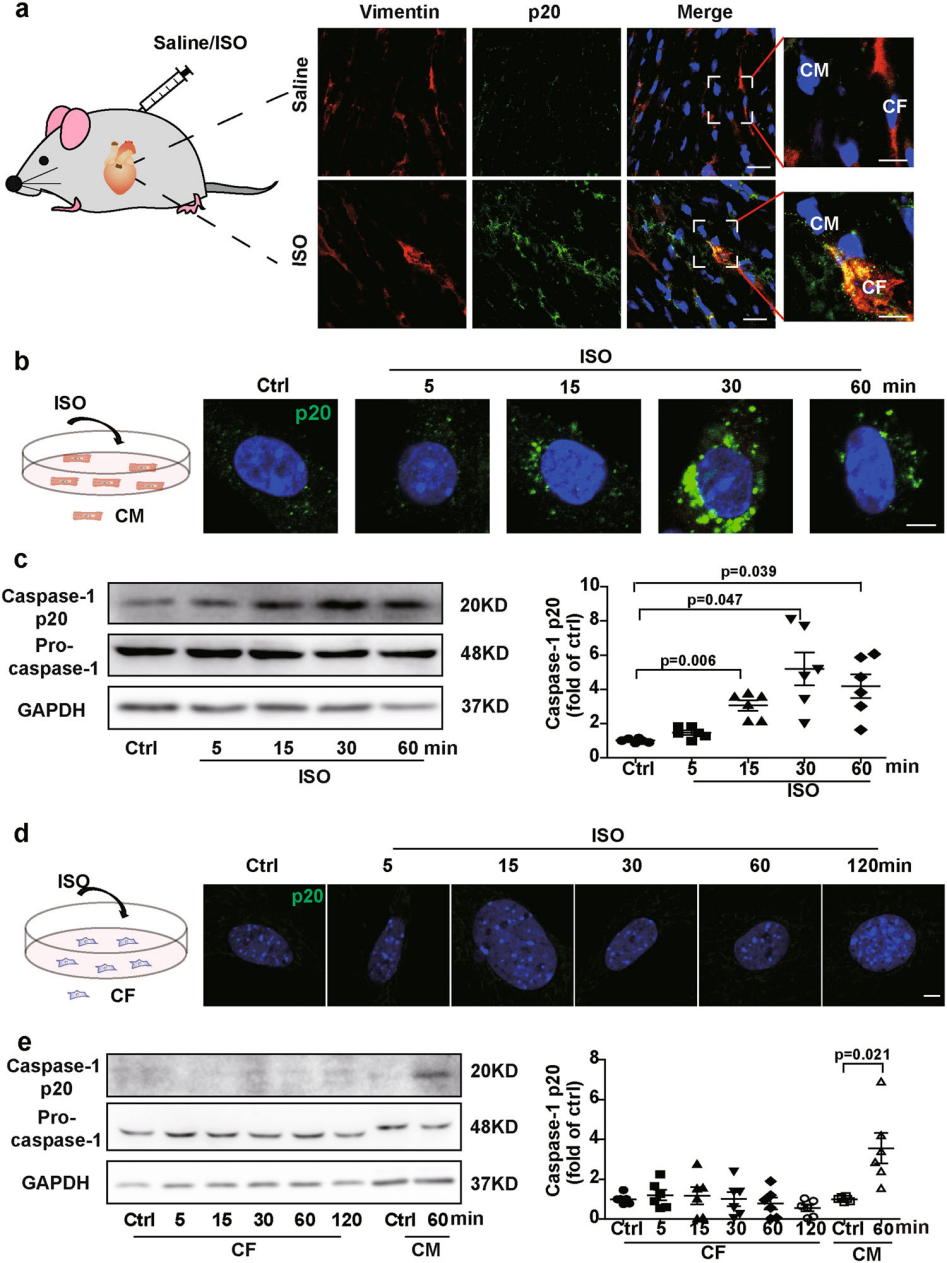
Upon ISO treatment, inflammasomes were activated in the cardiac fibroblasts (CFs) from heart tissue but not in the isolated neonatal CFs. **a** Cleaved caspase-1 (p20, green) was detected in the mouse heart tissue with immunofluorescence. The CFs were labeled with vimentin (red), a CF-specific marker. The nuclei were stained with Hoechst 33342 (blue). Scale bar: 20 μm. The scale bar of enlarged pictures was 5 μm. **b** Cleaved caspase-1 (p20, green) was detected in the isolated cardiomyocytes upon ISO treatment. The nuclei were stained with Hoechst 33342 (blue). Scale bar: 5 μm. **c** Western blotting analysis for pro-caspase-1 and cleaved caspase-1 (p20) in the cultured neonatal mouse cardiomyocytes was performed (*n* = 6). **d** Cleaved caspase-1 (p20, green) was detected in the neonatal mouse CFs upon ISO treatment. The nuclei were stained with Hoechst 33342 (blue). Scale bar: 5 μm. **e** Western blotting analysis for pro-caspase-1 and cleaved caspase-1 (p20) in the neonatal mouse CFs and cardiomyocytes was performed at the indicated time points after ISO treatment (*n* = 6). Data are presented as the mean ± SEM. Analyzed with Welch’s ANOVA with a Games-Howell post-hoc test or Welch’s *t*-test. Ctrl control, ISO isoproterenol, CM cardiomyocyte, CF cardiac fibroblast.

Immunofluorescence staining was used to detect inflammasome activation in the hearts of mice treated with a single dose of ISO for 1 h. The activated inflammasomes were labeled with caspase-1 (p20) antibody and appeared as perinuclear fluorescent specks^14^. As shown in Fig. 1a, ISO not only induced inflammasome activation in the cardiomyocytes but also in the CFs, which were identified by staining with the CF marker vimentin. Immunofluorescence staining and western blotting for caspase-1 were used to further evaluate the inflammasome activation in the isolated neonatal mouse cardiomyocytes and CFs. Caspase-1 cleavage significantly increased in the isolated cardiomyocytes 15 min after exposure to ISO and was sustained up to 1 h (Fig. 1b, c), suggesting that ISO can directly activate the inflammasomes in cardiomyocytes. However, ISO did not activate caspase-1 in the isolated neonatal mouse CFs (Fig. 1d, e). Therefore, the in vivo activation of inflammasomes in the CFs of mouse hearts may be due to the intercellular communication between cardiac cells.

### MNTs participated in the activation of inflammasomes in CFs

**Fig. 2:**
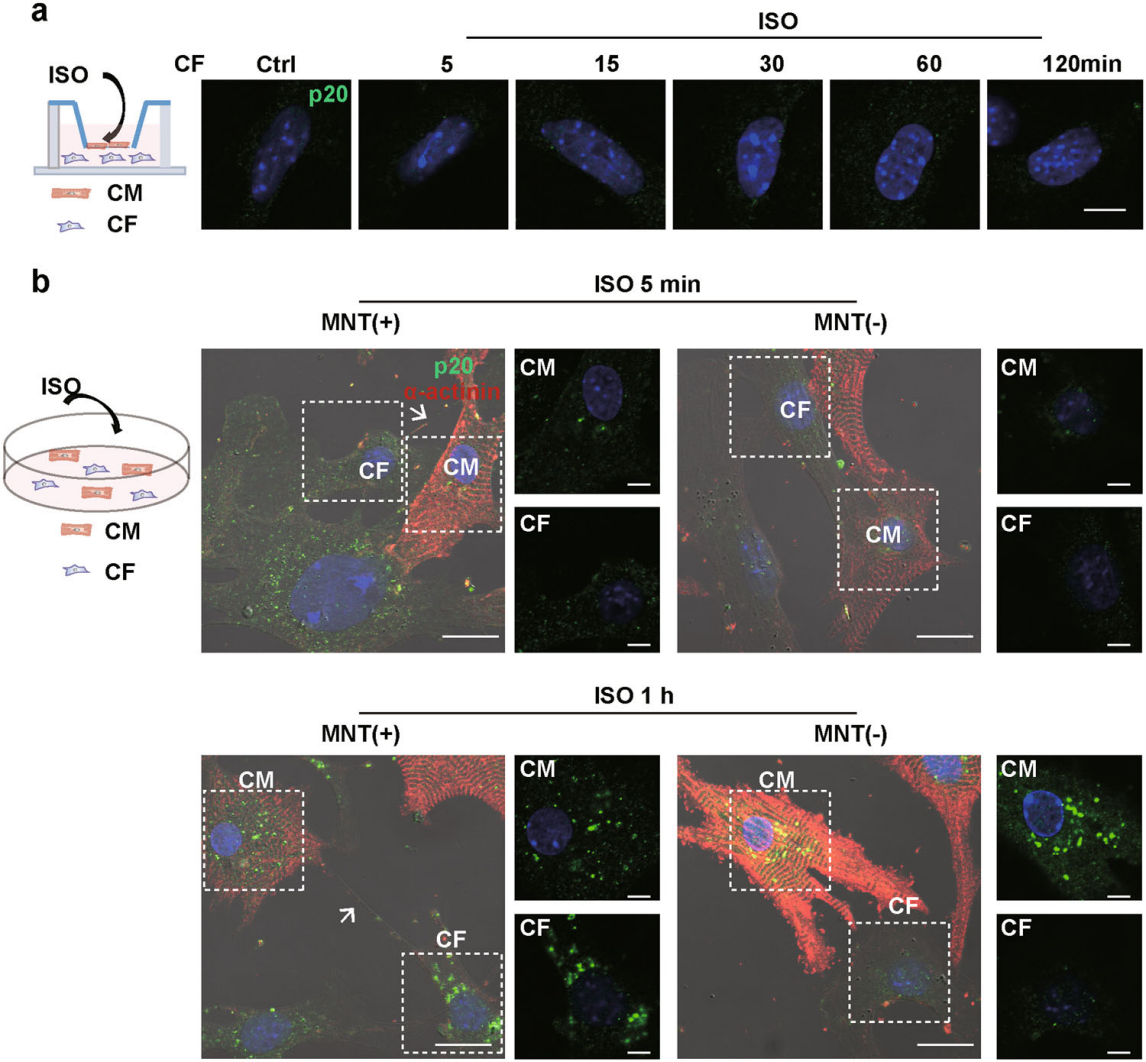
Upon β-AR overactivation, inflammasomes were activated in the CFs connected with cardiomyocytes via MNTs. **a** Cleaved caspase-1 (p20, green) was stained in the CFs cultured with cardiomyocytes in Transwell dishes, and the nuclei were stained with Hoechst 33342 (blue). Scale bar: 5 μm. **b** Cleaved caspase-1 (p20, green) was detected in the cocultured cardiomyocytes and CFs treated with ISO for 5 min and 1 h. Representative images show the cardiomyocytes and CFs connected with [MNT(+)] or without membrane nanotubes [MNT(−)]. The cardiomyocytes were stained with an antibody against cardiomyocyte-specific marker sarcomeric α-actinin (red). The nuclei were stained with Hoechst 33342 (blue). Scale bar: 20 μm. The scale bar of enlarged pictures was 5 μm. The white arrows pointed to the MNT. Ctrl control, ISO isoproterenol, CM cardiomyocyte, CF cardiac fibroblast.

To investigate whether cell-to-cell communication is involved in the inflammasome activation in CFs, cardiomyocytes and CFs were cultured in Transwell dishes, which enabled the cells to share the medium without direct contact. Caspase-1 was not increased in the CFs in the Transwell dishes following ISO treatment (Fig. 2a). Notably, when the cardiomyocytes and CFs were cocultured in the same dish, caspase-1 was increased and formed fluorescent specks in both cells treated with ISO for 1 h, but not 5 min (Fig. 2b and Fig. S1). This suggested that the inflammasome activation in the CFs requires direct contact between the cardiomyocytes and CFs. Furthermore, MNTs were formed between cardiomyocytes and CFs both in control and ISO-treated groups (Fig. S2). The caspase-1 was activated only in the CFs connected with cardiomyocytes via MNTs, but not in those without MNTs (Fig. 2b). In addition, ASC specks were detected in cardiomyocytes following ISO treatment, which further supports the activation of inflammasome (Fig. S3a, b). The ASC specks were also only formed in the MNT-connected CFs (Fig. S3c).

**Fig. 3:**
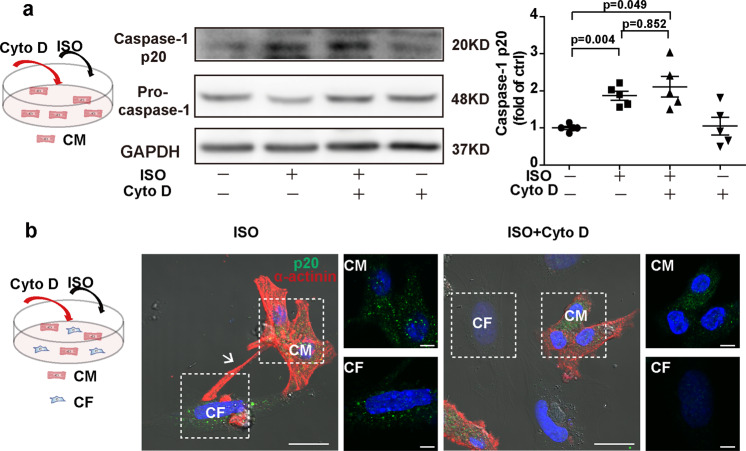
Disrupting MNTs inhibited inflammasome activation in the CFs cocultured with cardiomyocytes. **a** Cardiomyocytes were pretreated with or without the microfilament polymerization inhibitor cytochalasin D (Cyto D, 1 μM) for 4 h before exposure to ISO (10 μM) for 1 h. Western blotting analysis for pro-caspase-1 and cleaved caspase-1 (p20) in the cardiomyocytes was performed (*n* = 5). **b** The cocultured cardiomyocytes and CFs were pretreated with or without Cyto D for 4 h before exposure to ISO for 1 h. Cleaved caspase-1 (p20, green) was stained. Scale bar: 20 μm. The scale bar of enlarged pictures was 5 μm. The cardiomyocytes were stained with an antibody against cardiomyocyte-specific marker sarcomeric α-actinin (red). The nuclei were stained with Hoechst 33342 (blue). Data are presented as the mean ± SEM. Analyzed with Welch’s ANOVA with a Games-Howell post-hoc test. The white arrow pointed to the MNT. Ctrl control, ISO isoproterenol, CM cardiomyocyte, CF cardiac fibroblast.

To investigate the role of MNTs in the inflammasome activation of CFs, we disrupted the MNTs between the cocultured cardiomyocytes and CFs. Microfilaments are the integral structural component of MNTs, and an inhibitor of microfilament polymerization, cytochalasin D (Cyto D), disrupted the MNT structure (Fig. S4). Although the Cyto D treatment did not inhibit inflammasome activation in the cardiomyocytes (Fig. 3a), it inhibited inflammasome activation in the cocultured CFs (Fig. 3b and Fig. S5). To identify whether the signal transfer that induced inflammasome activation in CFs was dependent on microtubules, we applied the microtubule polymerization inhibitors nocodazole (Noc) and colcemid (Col). As shown in Fig. S6a, b, Noc and Col did not influence the formation of MNTs between cardiomyocytes and CFs but inhibited inflammasome activation in the cardiomyocytes treated with ISO (Fig. S6c, d). As a result, caspase-1 cleavage in the CFs was suppressed (Fig. S6e).

### NLRP3 in cardiomyocytes contributed to the activation of inflammasomes in CFs

**Fig. 4:**
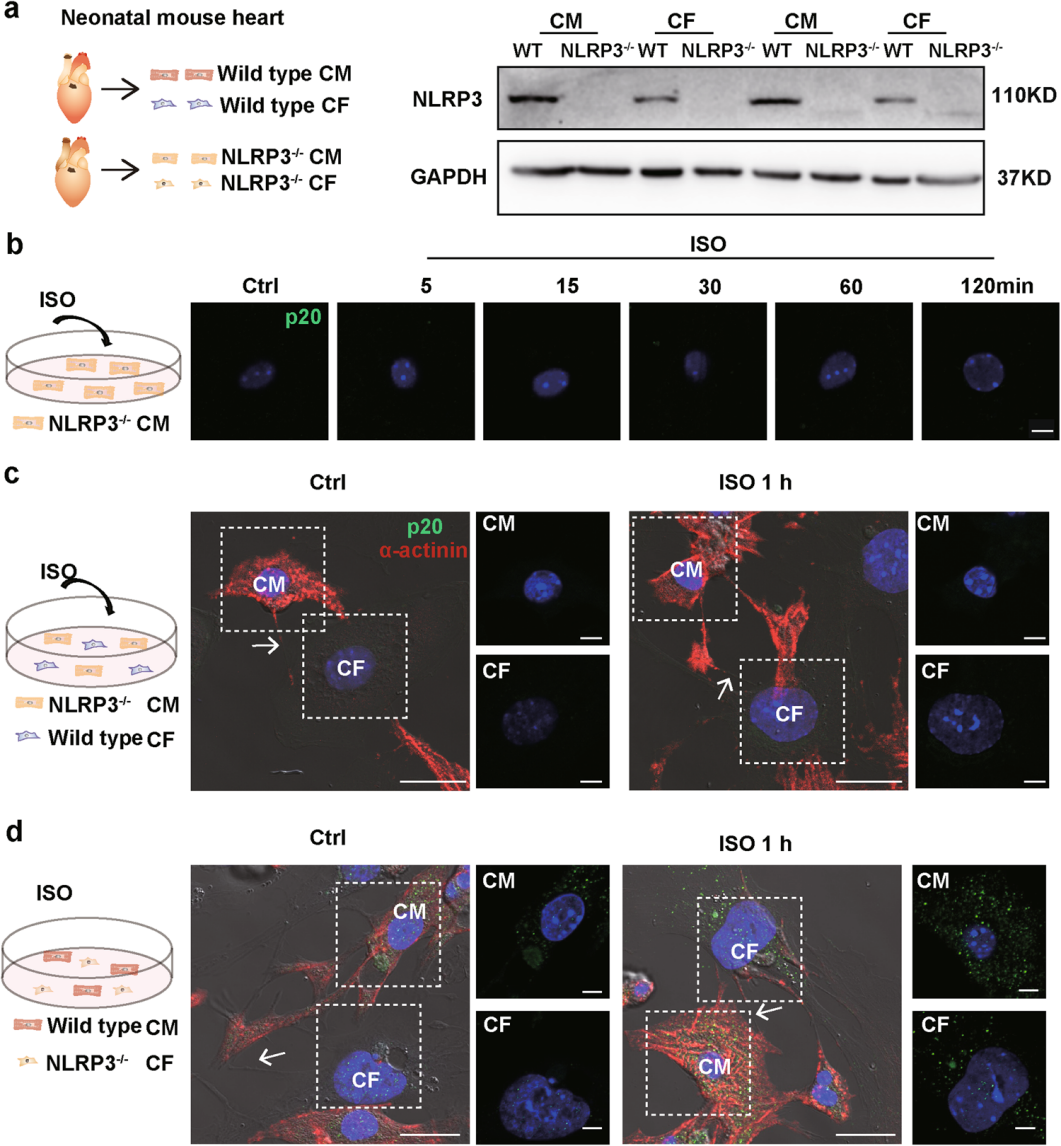
The activated inflammasome in the CFs was dependent on NLRP3 in the cardiomyocytes. **a** Western blot analysis for NLRP3 in the neonatal mouse cardiac cells of the wild-type (WT) or *Nlrp3*^−/−^ (KO) mice was performed. **b** Cleaved caspase-1 (p20, green) was detected in the *Nlrp3*^−/−^ cardiomyocytes upon ISO treatment. The nuclei were stained with Hoechst 33342 (blue). Scale bar: 5 μm. **c** Cleaved caspase-1 (p20, green) was immunostained in the cocultured *Nlrp3*^−/−^ cardiomyocytes and wild-type CFs upon ISO treatment. The images show the cardiomyocytes and CFs connected with membrane nanotubes. Cardiomyocyte markers (α-actinin, red) and nuclei (blue) were stained. Scale bar: 20 μm. The scale bar of enlarged pictures was 5 μm. The white arrows pointed to the MNT. **d** Immunostaining of cleaved caspase-1 (p20) in the ISO-treated cocultured wild-type cardiomyocytes and *Nlrp3*^−/−^ CFs was performed. Scale bar: 20 μm. The scale bar of enlarged pictures was 5 μm. The white arrows pointed to the MNT. Ctrl control, ISO isoproterenol, CM cardiomyocyte, CF cardiac fibroblast.

To identify the origin of the activated inflammasome in the CFs, we used NLRP3 knockout (*Nlrp3*^−/−^) neonatal mouse cardiomyocytes and CFs. First, we confirmed the depletion of NLRP3 in the cardiomyocytes and CFs by western blotting (Fig. 4a). ISO did not activate the inflammasomes in the *Nlrp3*^−/−^ cardiomyocytes (Fig. 4b). When the *Nlrp3*^−/−^ cardiomyocytes and wild-type CFs were cocultured, caspase-1 was not activated in the CFs, even when they were connected with cardiomyocytes via MNTs (Fig. 4c), which implied that inflammasome activation in the CFs was dependent on the activation of the NLRP3 inflammasome in the cardiomyocytes. In contrast, when the wild-type cardiomyocytes and *Nlrp3*^−/−^ CFs were cocultured, activated caspase-1 was detected in the CFs connected with cardiomyocytes via MNTs (Fig. 4d), suggesting that the NLRP3 in cardiomyocytes, but not in CFs, is necessary for the inflammasome activation in the cocultured CFs.

### CF pyroptosis relied on activated inflammasomes transferred from cardiomyocytes via MNTs

**Fig. 5:**
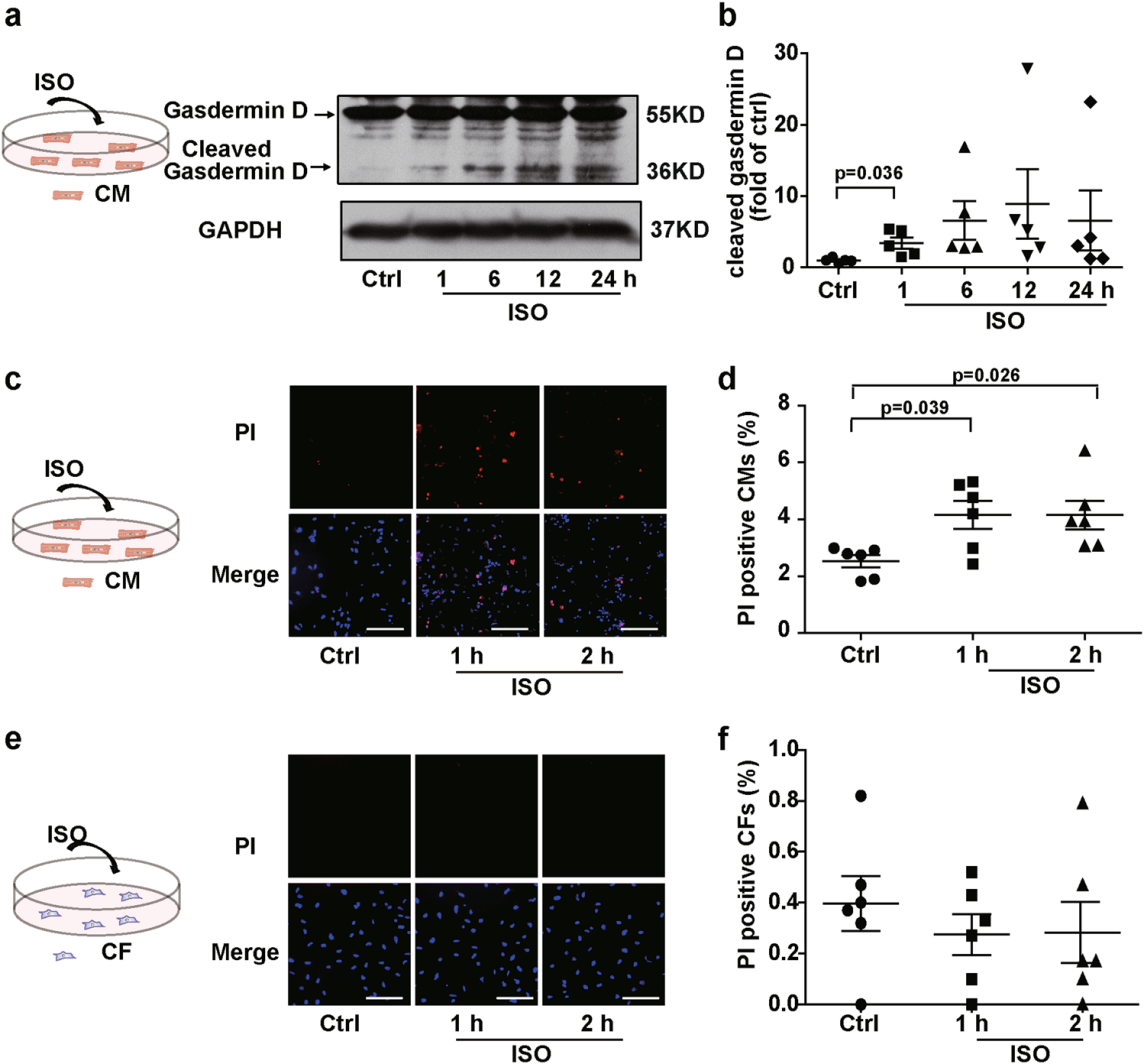
ISO induced pyroptosis in the cardiomyocytes but not the CFs. **a**, **b** Western blotting analysis for gasdermin D and cleaved gasdermin D in the cardiomyocytes was performed at the indicated time points following ISO treatment (*n* = 5). **c**, **d** Cardiomyocyte pyroptosis upon ISO treatment was determined with PI staining and imaged and quantified by high-content screening (*n* = 6). **e**, **f** CF pyroptosis upon ISO treatment was determined with PI staining and quantified by a high-content screening platform (*n* = 6). Scale bar: 100 μm. Data are shown as the mean ± SEM. Analyzed with Kruskal–Wallis ANOVA combined with post-hoc Dunn’s multiple comparison.

Activated caspase-1 cleaves gasdermin D, whose N-terminal transfers to the plasma membrane and causes cell pyroptosis^15^. Consistent with inflammasome activation, ISO induced gasdermin D cleavage (Fig. 5a, b) and positive PI staining in the isolated cardiomyocytes at 1 h (Fig. 5c, d), indicating that ISO induced pyroptosis at the early stage of ISO treatment. In contrast, the western blotting results showed that ISO did not induce caspase-3 cleavage until 12 h (Fig. S7a, b), and the TUNEL-positive cardiomyocytes were not increased until 24 h after ISO treatment (Fig. S7c, d). These findings indicated that ISO treatment caused rapid pyroptosis but not apoptosis in cardiomyocytes in the early phase (within 24 h).

**Fig. 6:**
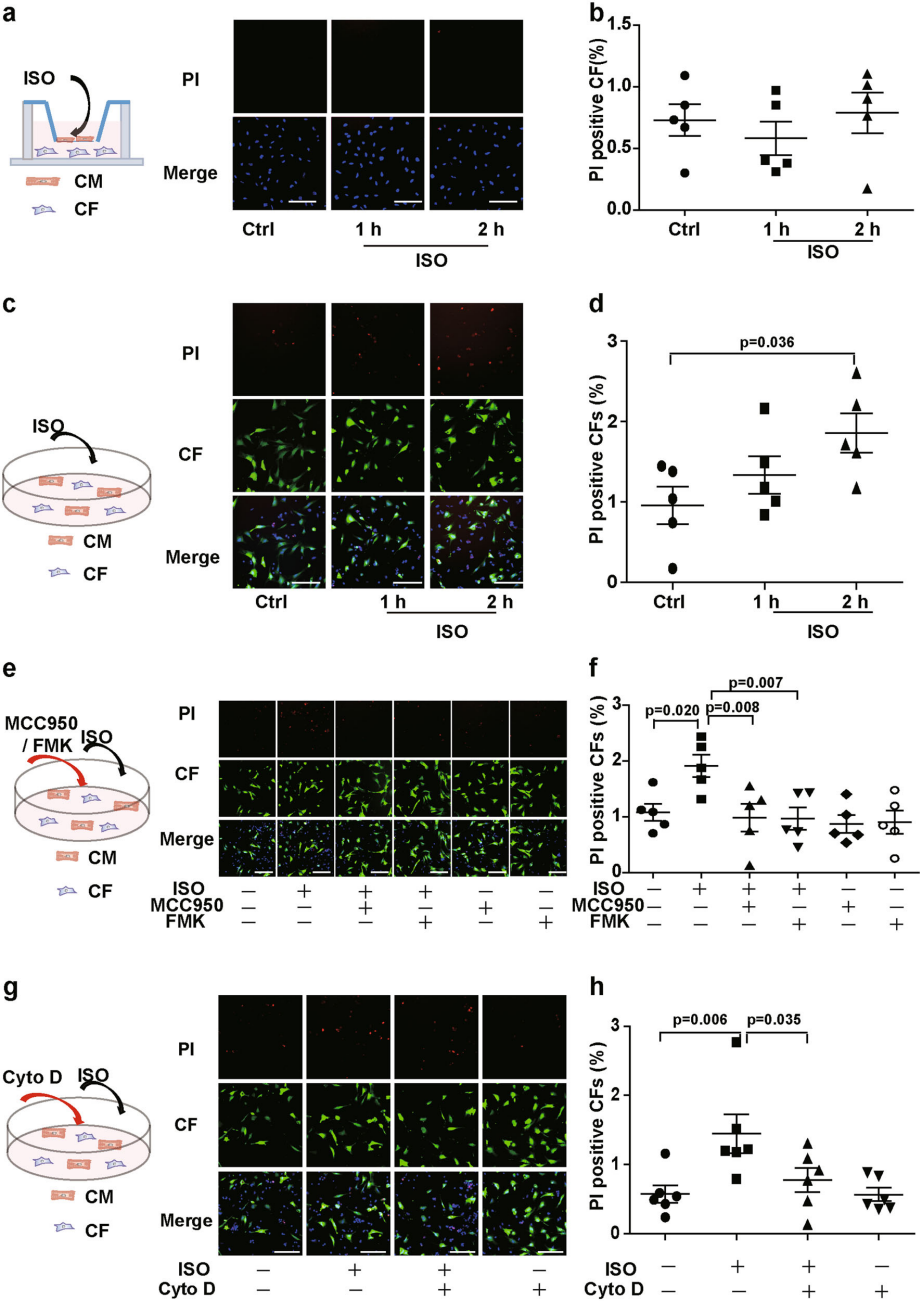
ISO induced pyroptosis in the CFs that connected with cardiomyocytes via MNTs. The CFs were labeled with CellTracker Green (green) and then transferred into the dishes with cardiomyocytes. Pyroptosis was detected after ISO treatment by PI staining (red) and quantified by a high-content screening platform. **a**, **b** Pyroptosis of the CFs cocultured with cardiomyocytes in Transwell dishes following ISO treatment was detected (*n* = 5). **c**, **d** Pyroptosis of the CFs directly cocultured with cardiomyocytes following ISO treatment was detected (*n* = 5). **e**, **f** Pyroptosis of the CFs cocultured with cardiomyocytes following ISO treatment was detected. The cells were pretreated with the inflammasome inhibitors MCC950 (1 μM) and Z-YVAD-FMK (FMK, 2 μM) for 30 min (*n* = 5). **g**, **h** Pyroptosis of the CFs cocultured with cardiomyocytes following ISO treatment was detected. The cells were pretreated with Cyto D for 4 h (*n* = 5). Scale bar: 100 μm. Data are shown as the mean ± SEM. Analyzed with one-way ANOVA with Bonferroni’s post-hoc test.

Consistent with inflammasome activation, ISO did not directly induce pyroptosis in the isolated CFs (Fig. 5e, f) or in the CFs cultured with cardiomyocytes in Transwell dishes (Fig. 6a, b). When coculturing CFs with cardiomyocytes, ISO induced the pyroptosis of CFs that were connected to cardiomyocytes with MNTs (Fig. S8). We used a high-content screening platform to quantify the ratio of pyroptotic CFs and found that ISO increased pyroptosis in the CFs cocultured with cardiomyocytes (Fig. 6c, d). The pyroptosis of CFs was suppressed by the inhibition of inflammasome activation with the NLRP3 inhibitor MCC950 and the caspase-1 inhibitor Z-YVAD-FMK (Fig. 6e, f). MCC950 also inhibited the pyroptosis of *Nlrp3*^−/−^ CFs when coculturing with wild-type cardiomyocytes, which suggests the cardiomyocytes-derived NLRP3 was involved in the pyroptosis of CFs (Fig. S9). Furthermore, to clarify the role of MNTs in the pyroptosis of the CFs cocultured with cardiomyocytes, we used Cyto D to disrupt the MNT structure. Cyto D inhibited the pyroptosis of the cocultured CFs upon ISO treatment (Fig. 6g, h), which suggests that MNTs mediated the ISO-induced pyroptosis of CFs when cocultured with cardiomyocytes.

## Discussion

**Fig. 7:**
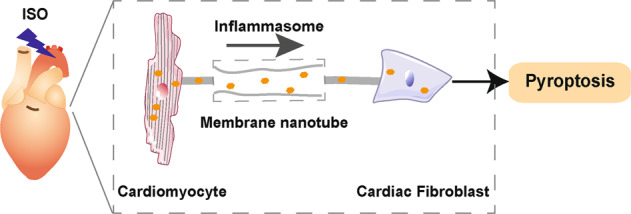
Schematic of MNT-mediated transfer of the inflammasome between cardiac cells upon sympathetic stress. In the early phase of acute β-AR overactivation, inflammasomes are activated in cardiomyocytes and transferred to CFs via MNTs, which results in pyroptosis of the CFs.

Our study demonstrated that at the early stage of acute β-AR overactivation, MNTs acted as the “highway” between cells and mediated the spread of inflammasome activation from cardiomyocytes to CFs. As a result, the activated inflammasomes induced pyroptosis of CFs (Fig. 7). This finding suggests that MNTs contribute to the rapid spread of inflammatory molecules among cardiac cells upon β-AR overactivation; inhibiting inflammasome propagation can be a potential therapeutic strategy for the treatment of cardiac inflammatory injuries.

Inflammation is a well-known feature of many cardiovascular diseases and is dependent on the various types of cell-to-cell communication between multiple cells for the generation of the inflammatory cascade. Lipopolysaccharide (LPS)-stimulated monocytic cells release extracellular vesicles to induce an inflammatory response in endothelial cells^16^. Homocysteine-treated adipocytes activate macrophage NLRP3 inflammasomes in adipose tissue in a paracrine manner^17^. Similarly, our previous study found that β-AR insult causes inflammasome activation in cardiomyocytes, which initiates cardiac inflammation by promoting cytokine release and intercellular communications among cardiomyocytes, CFs, and macrophages^5^. In addition to the release of cytokines, MNTs mediated the rapid direct transfer of inflammasomes and intracellular inflammatory molecules as we showed in the present study. Moreover, MNTs have been found between immune cells^18^. Hence, MNTs may form an intercellular network for the rapid direct communication of inflammatory molecules between cardiac cells and immune cells, which may be a new mechanism of inflammatory progression besides the cytokine network.

MNTs enable the rapid transfer of intracellular molecules between distant cells. In a cerebrospinal fluid-cultured cell model, the “prion-like propagation” of transactive response DNA-binding protein of 43 kDa (TDP-43) relied on the MNT-like structure but not exosomes^19^. In the present study, β-AR induced the activation of the inflammasome in CFs via MNTs within 1 h, both in vivo and in vitro. The rapid propagation of inflammasome activation via MNTs may be important for the amplification of the inflammatory cascade, especially in the early initiation of cardiac inflammation following ISO treatment.

Our previous study demonstrated that the MNTs between cardiomyocytes and CFs contain microfilaments and partially contain microtubules that mediate mitochondrial transfer^13^. Microfilaments are the basic component of MNTs. Here, we found that a microfilament inhibitor disrupted the MNTs and inhibited the propagation of inflammasome activation into the CFs. In contrast, the microtubule depolymerization drugs Noc and Col directly suppressed inflammasome activation in cardiomyocytes. Microtubules and motor proteins, such as dynein and kinesin, have been reported to enable the necessary spatial arrangement of the components of the NLRP3 inflammasome in the cell, leading to inflammasome activation^20–22^. The activation of inflammasomes under acute sympathetic stimulation may also depend on microtubules. Therefore, upon β-AR overactivation, microfilaments and microtubules play different roles in the propagation of inflammation: microfilaments mediate the inflammasome transfer via MNTs, and microtubules directly participate in inflammasome activation.

MNTs are bridges that transfer various cargoes. Inflammasomes are protein complexe. NLRP3 is an important component of inflammasomes. Our previous study revealed that ISO increased the expression of NLRP3 in cardiomyocytes^5^. The present study further found that the knockout of NLRP3 in cardiomyocytes suppressed the caspase-1 cleavage and pyroptosis of the cocultured CF following ISO treatment. Whereas, ISO activated the inflammasomes in the MNT-connected *Nlrp3*^−/−^ CFs cocultured with the wild-type cardiomyocytes. It suggests the NLRP3 in cardiomyocytes is necessary for the MNT-mediated activation of inflammasome in both wild-type and *Nlrp3*^−/−^ CFs. This finding also implies that MNTs may transfer activated inflammasome complexes or inflammasome activators from cardiomyocytes into CFs resulting in the activation of inflammasomes in the CFs.

Similarly, Esposti et al. found that upon stimulation with Fas, activated caspase-3 in T cells was transported to unstressed cells through MNTs, which led to apoptosis^23^. Therefore, MNTs could transfer activated caspase family proteins to execute a physiological function. Current research has mainly focused on apoptotic signals that diffuse within or between cells. Ferrel et al. revealed the mechanism underlying the propagation of apoptosis through the cytoplasm: once death signals appear, the triggering wave in the cell will be transmitted to every corner of the cell, which leads to the death of the whole cell^24^. Esposti et al. also found that apoptotic cells stimulated by Fas transferred membrane components via MNTs to contribute to the apoptosis of recipient cells^23^. Although apoptosis has been reported to diffuse among cells, it is unclear whether other types of cell death can also spread to other cells. The present study showed that cardiomyocytes transferred inflammasomes to CFs through MNTs, resulting in pyroptosis. Thus, our study provides evidence that pyroptosis can also diffuse between cells.

Pyroptosis is a form of lytic programmed cell death initiated by inflammasomes. It is known as “programmed cell necrosis” and plays a proinflammatory role through rapid membrane disruption and the release of inflammatory mediators^25^. Following myocardial infarction, pyroptotic cardiomyocytes released various proinflammatory factors^26^. Pyroptotic endothelial cells secrete proinflammatory factors such as IL-1β and IL-18^27^. The released proinflammatory factors following pyroptosis further act on other cells and lead to the activation of the inflammatory cascade^28^. Therefore, ISO stimulation leads to rapid pyroptosis of cardiomyocytes and CFs, which can further promote inflammation. The transfer of inflammasomes via MNTs may expand the area of pyroptosis, aggravating inflammatory progression.

In conclusion, MNTs facilitate the spread of inflammasome activation from cardiomyocytes to CFs following β-AR overactivation, causing the pyroptosis of CFs. This study highlights the important role of MNTs in the propagation of inflammatory injury upon β-AR insult. Therefore, preventing inflammasome transfer is a potential therapeutic strategy for alleviating acute β-AR overactivation-induced cardiac injury.

## Materials and methods

### Animals and drug treatment

All experimental procedures and animal protocols were approved by the Committee of Peking University on the Ethics of Animal Experiments (LA2016-018) and conducted in accordance with the Guide for the Care and Use of Laboratory Animals published by the US National Institutes of Health (NIH Publication no. 85-23, revised 2011).

Wild-type male C57BL/6N mice (10 weeks old) were provided by the Animal Department of Peking University Health Science Center (Beijing). The *Nlrp3*^*−*^^/−^ mice were from The Jackson Laboratory (Stock No. 017969, Strain Name B6.129-*Nlrp3*^*tm1Hhf*^/J). Mice were randomly grouped and subcutaneously injected with a single dose of ISO (Sigma-Aldrich, St. Louis, MO, USA; 5 mg/kg body weight) or saline for 1 h. The sample size estimation was not adopted as the samples were not for quantitively statistical analysis.

### Frozen sections of heart tissue

After ISO treatment for 1 h, the mice were anesthetized with 3% isoflurane in oxygen, and the pedal pinch reflexes were completely inhibited before euthanasia. The heart tissues were then isolated and fixed with optimal cutting temperature compound (4583, SAKURA, Torrance, CA, USA). Frozen sections of the mouse hearts (6-μm thick) were obtained using a cryostat (Leica, Wetzlar, Germany), placed on poly-L-lysine-coated glass slides, soaked with acetone, permeabilized with 0.2% Triton X-100 in phosphate-buffered saline (PBS) for 10 min, blocked with 5% BSA and then stained with primary antibodies against vimentin (1:100 dilution, ab8979, Abcam, Cambridge, MA, USA), cleaved caspase-1 p20 (1:200 dilution, PRS3459, Sigma-Aldrich), and the nuclei dye Hoechst 33342. The frozen sections were visualized using a laser scanning confocal microscope (LMS 780, Carl Zeiss, Inc., Thornwood, NY, USA) with a 63×/1.4NA oil immersion objective lens and an excitation wavelength of 405/488/543 nm. All images were acquired with ZEN 2012 software. The immunofluorescence staining and image capturing were carried out without knowledge of treatments.

### Primary cell isolation and culture

Neonatal mouse cardiomyocytes and CFs were isolated and cultured as previously described^29^. Briefly, the hearts of 1 day-old C57BL/6 (provided by the Animal Department of Peking University) and NLRP3 knockout mice were digested with trypsin (27250-018, Gibco, Carlsbad, CA, USA) and collagenase II (17101-015, Gibco) at 37 °C. The isolated cells were plated on 10 cm dishes in minimum essential medium with 10% fetal bovine serum for 2 h to allow the CFs to attach. The unattached cardiomyocytes were then transferred to new culture dishes for subsequent treatment. The CFs and cardiomyocytes were cultured at 37 °C in humidified air containing 5% CO_2_.

### Immunofluorescence

For immunofluorescence staining, the cells were fixed with 4% paraformaldehyde for 15 min in PBS, permeabilized with 0.2% Triton X-100 in PBS, blocked with blocking buffer and then stained with primary antibodies against α-tubulin (ab52866, Abcam), α-actinin (A7811, Sigma-Aldrich), or cleaved caspase-1 p20 (PRS3459, Sigma-Aldrich) followed by Alexa Fluor 488/546/633 secondary antibodies. ASC was detected with an Alexa Fluor 488 Conjugated antibody (17507, Cell Signaling). Rhodamine phalloidin dye (R415, Invitrogen, Carlsbad, CA, USA) was incubated with the cells for 20 min at 37 °C for F-actin. The stained cells were visualized and analyzed using a laser scanning confocal microscope with a 63×/1.4NA oil immersion objective lens and excitation wavelengths of 488, 543, and 633 nm. All images were acquired using ZEN 2012 software.

### Western blotting

The cells were lysed (1% deoxycholic acid, 10 mM Na_4_P_2_O_7,_ 1% Triton 100, 10% glycerol, 100 mM NaCl, 5 mM EDTA, 20 mM Tris-HCl, 0.1% SDS, 50 mM NaF, 1 mM Na_3_VO_4_), and a BCA Protein Assay kit (23228; 23224, Thermo Fisher Scientific, Rockford, IL, USA) was used to measure the protein concentrations. Samples containing 40–60 μg of protein were electrophoresed by 12% sodium dodecyl sulfate polyacrylamide gel electrophoresis (SDS-PAGE) and transferred to polyvinylidene fluoride membranes. After the membranes were blocked with 5% nonfat milk in TBST, they were incubated with the appropriate primary antibodies against cleaved caspase-1 p20 (1:1000 dilution, PRS3459, Sigma-Aldrich), pro-caspase-1 (1:1000 dilution, 3866S, Cell Signaling Technology Incorporated, Danvers, MA, USA), gasdermin D (1:1000 dilution, ab209845, Abcam), NLRP3 (1:1000 dilution, 15101, Cell Signaling Technology Incorporated, Danvers, MA, USA) or GAPDH (1:10,000 dilution, 2118 S, Cell Signaling Technology Incorporated) overnight at 4 °C, followed by secondary antibodies (1:2000 dilution for cleaved caspase-1 and gasdermin D, 1:3000 dilution for pro-caspase-1, 1:20,000 dilution for GAPDH) for 1 h at room temperature.

### Cell death detection

Apoptosis was detected by TUNEL assays (G3250, Promega Corporation, Fitchburg, WI, USA). The cell nuclei were stained with Hoechst 33342 (H3570, Invitrogen). TUNEL-positive cells were quantified by a high-content screening platform (Cellomics ArrayScan Infinity, Thermo Fisher Scientific, Rockford, IL, USA) in 20 randomly chosen sites in every well of the cell culture plate. Pyroptosis was determined by staining with propidium iodide (PI, P3566, Invitrogen) and quantified by the high-content screening platform.

### Statistical analysis

Data are represented as the mean ± standard error of the mean (S.E.M.) of at least four independent experiments. Statistical analyses were performed using GraphPad Prism 5.0 (GraphPad Software, Inc., La Jolla, CA, USA) and IBM SPSS Statistics 21.0 (IBM Corporation, Armonk, NY, USA). Sample size estimation was performed with PS Power and Sample Size Calculations program version 3.0. Data analyses were carried out without knowledge of treatments (blinded assessment). For parametric data, normality were tested with K–S test. If data were normally distributed. Two-tailed student’s *t*-test or analysis of variance (ANOVA) combined with Bonferroni’s post-hoc test was used to analyze the differences among groups for data with equal variances. For data with unequal variances, Welch’s *t*-test and Welch’s ANOVA with a Games–Howell post-hoc test were used. For nonparametric data, the Mann–Whitney *U*-test with the exact method was used to analyze the differences between two groups. A Kruskal–Wallis ANOVA combined with post-hoc Dunn’s multiple comparison test was performed when more than two groups were evaluated. Differences were considered significant at *P* < 0.05.

## Supplementary information

Supplementary Figure Legends

Figure S1

Figure S2

Figure S3

Figure S4

Figure S5

Figure S6

Figure S7

Figure S8

Figure S9
