# Supplementary material for: Membrane nanotubes facilitate the propagation of inflammatory injury in the heart upon overactivation of the β-adrenergic receptor
Source: Cell Death Dis. 2020 Nov 7;11(11):958. doi: 10.1038/s41419-020-03157-7 (PMC7648847; doi:10.1038/s41419-020-03157-7)
Supplement: Supplementary file 1 — Supplementary Figure Legends [file 41419_2020_3157_MOESM1_ESM.pdf]

## Supplementary Figure Legends

**Figure S1.** Inflammasome detection in the cocultured cardiomyocytes and CFs **(**the control group of Figure 2b). Cleaved caspase-1 (p20, green) was detected in the cocultured cardiomyocytes and CFs. The cardiomyocytes were stained with an antibody against cardiomyocyte-specific marker sarcomeric α-actinin (red). The nuclei were stained with Hoechst 33342 (blue). Scale bar: 20 μm. The scale bar of enlarged pictures was 5 μm. Ctrl, control; CM, cardiomyocyte; CF, cardiac fibroblast.

**Figure S2.** The representative images and numbers of MNTs between CMs and CFs. Cell membrane was stained with WGA488 (green). The cardiomyocytes were stained with an antibody against cardiomyocyte-specific marker sarcomeric α-actinin (red). White arrow indicates the MNT between CMs and CFs. Scale bar: 20 μm. Ctrl, control; CM, cardiomyocyte; CF, cardiac fibroblast; MNT: membrane nanotube. (n = 5) Two-tailed students t -test was used.

**Figure S3.** The ASC specks in isolated cardiac cells following ISO treatment. (a) ASC (green) in the isolated cardiomyocytes upon ISO treatment for different durations. The nuclei were stained with Hoechst 33342 (blue). Scale bar: 5 μm. (b) ASC (green) in the isolated cardiomyocytes upon ISO treatment with or without pretreatment with propranolol (Prop). The nuclei were stained with Hoechst 33342 (blue). Scale bar: 5 μm. (c) ASC specks (green) in the cocultured cardiomyocytes and CFs. Representative images show the cardiomyocytes and CFs connected with [MNT(+)] or without membrane nanotubes [MNT(-)]. The cardiomyocytes were stained with an antibody against cardiomyocyte-specific sarcomeric α-Actinin (red). The nuclei were stained with Hoechst 33342 (blue). Scale bar: 20 μm.

**Figure S4.** Microfilament is essential for the membrane nanotubes (MNTs) between neonatal mouse cardiomyocytes and CFs. (a) The cell membrane was labeled with the membrane dye WGA

Alexa Fluor 488 conjugate (WGA, green), F-actin (component of microfilament) was labeled with rhodamine phalloidin (red), and the cardiomyocytes were stained for α-actinin (purple). The white arrows indicated the MNTs. Scale bar: 10 μm. (b) MNT structure was disrupted by microfilament polymerization inhibitor. After being treated with microfilament polymerization inhibitor, cytochalasin D (Cyto D), the cells were labeled for microtubules and microfilament with α-tubulin antibodies (blue) and rhodamine phalloidin (F-actin, red), respectively. The white arrows pointed to the MNT. Scale bar: 20 μm. CM, cardiomyocyte; CF, cardiac fibroblast.

**Figure S5.** Inflammasome detection in the cocultured cardiomyocytes and CFs **(**the control group of Figure 3b). Cleaved caspase-1 (p20, green) was detected in the cocultured cardiomyocytes and CFs. The cardiomyocytes were stained with an antibody against cardiomyocyte-specific marker sarcomeric α-actinin (red). The nuclei were stained with Hoechst 33342 (blue). The white arrows pointed to the MNT. Scale bar: 20 μm. The scale bar of enlarged pictures was 5 μm. Ctrl, control; CM, cardiomyocyte; CF, cardiac fibroblast.

**Figure S6.** Disrupting microtubules inhibited the inflammasome activation in the cardiomyocytes and CFs upon ISO treatment. (a) The cocultured cardiomyocytes and CFs were labeled by WGA (green), α-tubulin (blue), and F-actin (red), and then treated with or without the microtubule depolymerization reagents nocodazole (Noc) and colcemid (Col). The white arrows pointed to the MNT. Scale bar: 20 μm. (b) Quantification of 40 randomly selected fields showed no difference in the number of MNTs between the cardiomyocytes and CFs in the control, Noc, and Col treatment groups (n = 4). (c, d) Cardiomyocytes were pretreated with or without the microtubule depolymerization drugs Noc and Col for 4 h before exposure to ISO (10 μM). Western blot analysis for pro-caspase-1 and cleaved caspase-1 (p20) was performed in the cardiomyocytes (n = 5). Ctrl: control. (e) The cocultured cardiomyocytes and CFs were pretreated with or without Noc and Col

for 4 h before exposure to ISO, and cleaved caspase-1 (green) was stained. The cardiomyocytes were stained with an antibody against cardiomyocyte-specific marker sarcomeric α-actinin (red). The nuclei were stained with Hoechst 33342 (blue). Scale bar: 20 μm. The scale bar of enlarged pictures was 5 μm. The white arrows pointed to the MNT. Data are presented as the mean ± SEM. One-way ANOVA with Bonferronis post hoc test.

**Figure S7.** ISO induced apoptosis in the cardiomyocytes. (a) and (b) Western blot analysis for pro-caspase-3 and cleaved caspase-3 was performed in the isolated neonatal mouse cardiomyocytes. (n = 5) Ctrl: control. (c) and (d) ISO-induced apoptotic cardiomyocytes were identified by TUNEL staining (green) and high-content screening imaging (n = 4). Scale bar: 100 μm. Data are presented as the mean ± SEM. Kruskal–Wallis ANOVA combined with a post hoc Dunn's multiple comparison test.

**Figure S8.** ISO induced the pyroptosis of MNT-connected CFs. CFs were stained with celltracker green and then cocultured with cardiomyocytes. The PI was stained to show the pyroptosis (red) in the cocultured cardiomyocytes and CFs. The white arrows pointed to the MNT. Scale bar: 20 μm. Ctrl, control; CM, cardiomyocyte; CF, cardiac fibroblast; MNT: membrane nanotube.

**Figure S9.** MCC950 inhibited the pyroptosis of *Nlrp3*$^{-/-}$ CFs cocultured with wild-type cardiomyocytes. CFs were isolated from *Nlrp3*$^{-/-}$ mice. The cells were stained with celltracker green and cocultured with wild-type CMs. ISO-induced pyroptosis of CFs was identified by PI staining (red) and high-content screening imaging (n = 6). Scale bar: 100 μm. Data are presented as the mean ± SEM. One-way ANOVA combined with a post hoc Bonferronis multiple comparison test.
