# Supplementary figures and images for: Membrane nanotubes facilitate the propagation of inflammatory injury in the heart upon overactivation of the β-adrenergic receptor
Source: Cell Death Dis. 2020 Nov 7;11(11):958. doi: 10.1038/s41419-020-03157-7 (PMC7648847; doi:10.1038/s41419-020-03157-7)

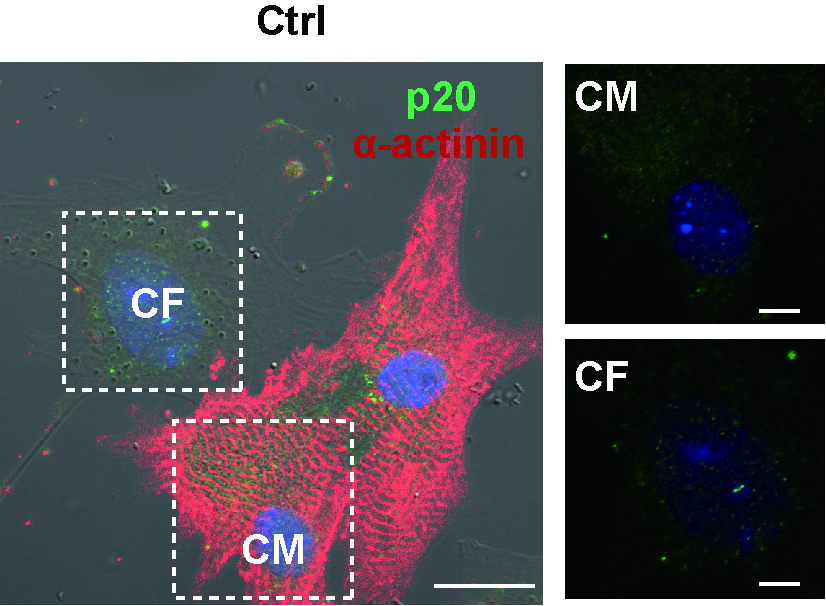

Supplement: Supplementary file 2 — Figure S1 [file 41419_2020_3157_MOESM2_ESM.tif]

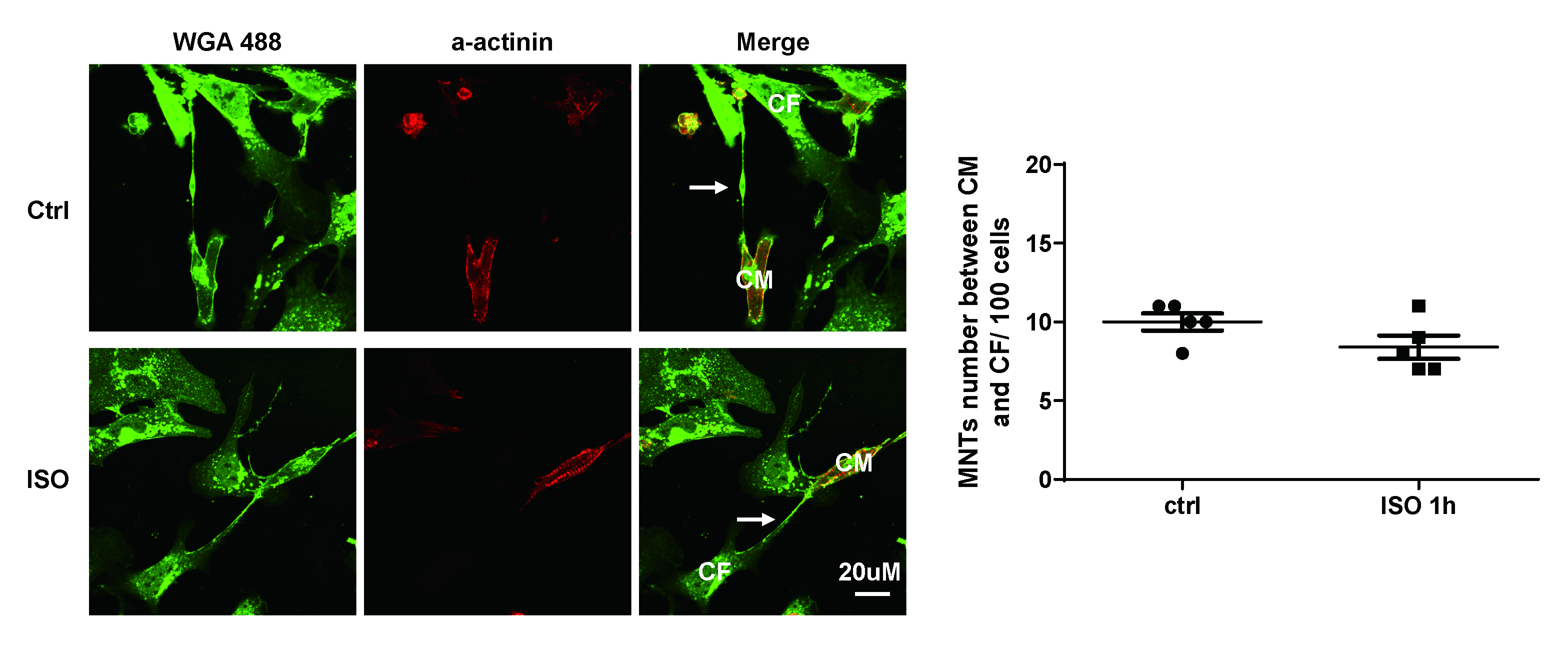

Supplement: Supplementary file 3 — Figure S2 [file 41419_2020_3157_MOESM3_ESM.tif]

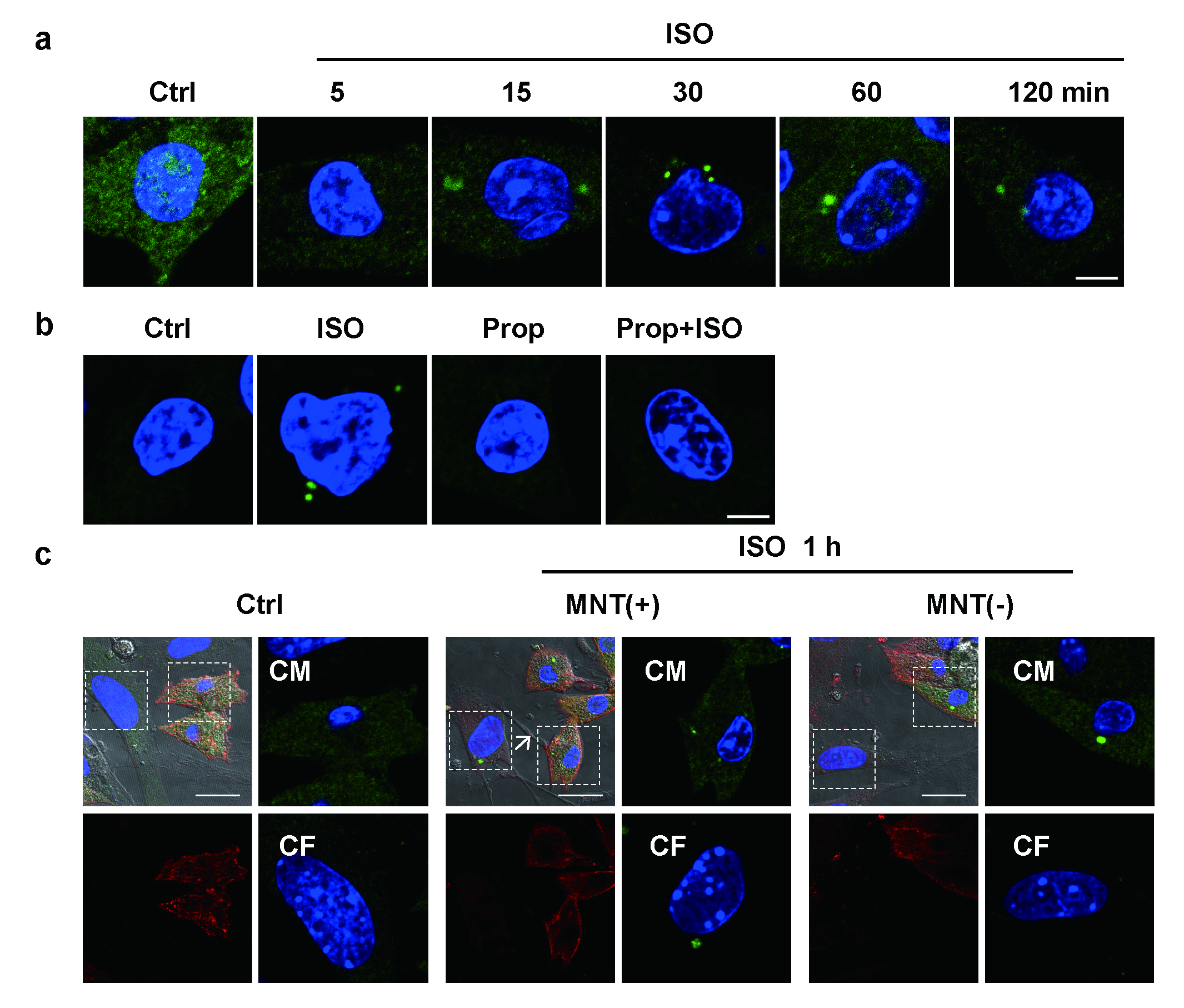

Supplement: Supplementary file 4 — Figure S3 [file 41419_2020_3157_MOESM4_ESM.tif]

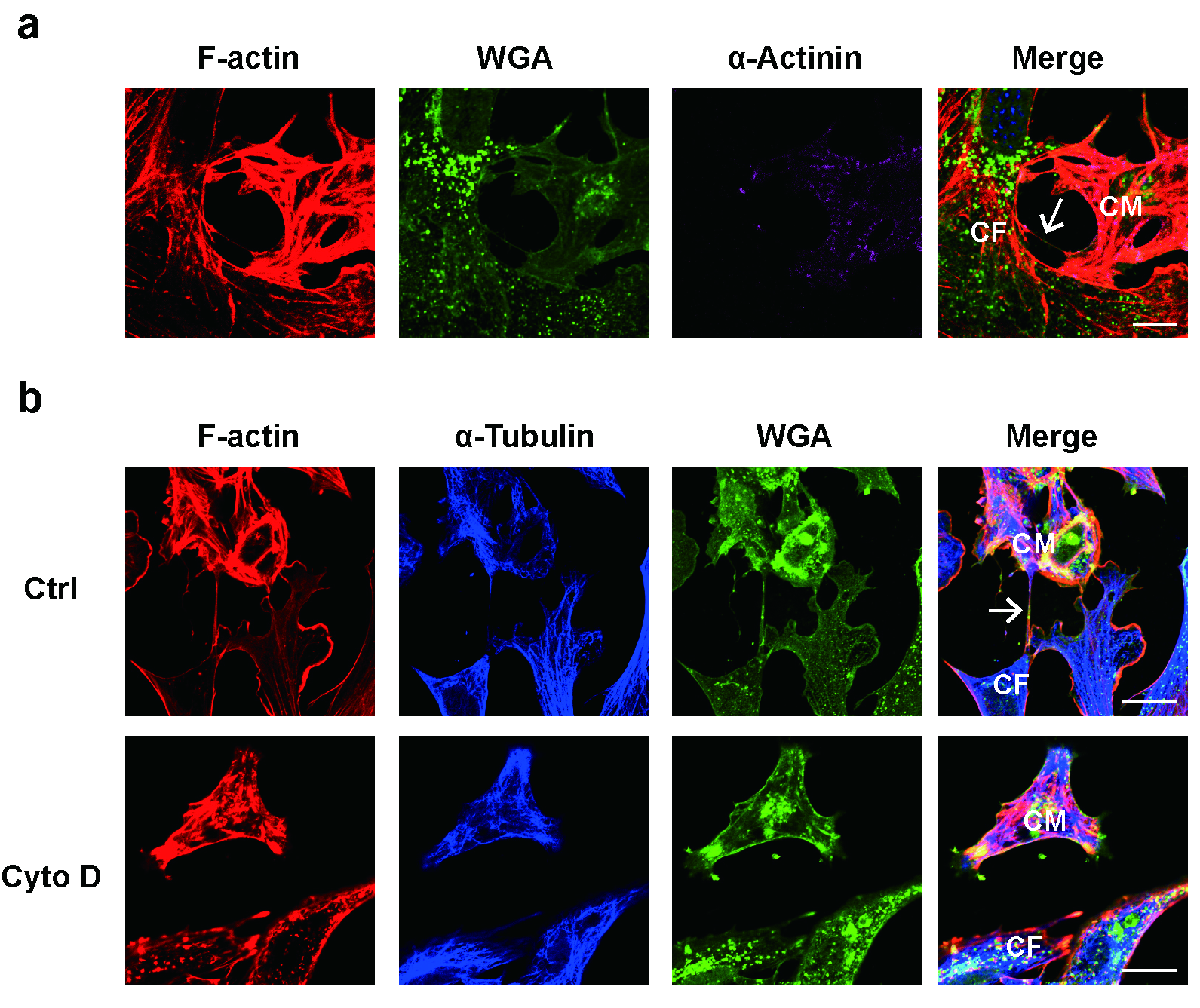

Supplement: Supplementary file 5 — Figure S4 [file 41419_2020_3157_MOESM5_ESM.tif]

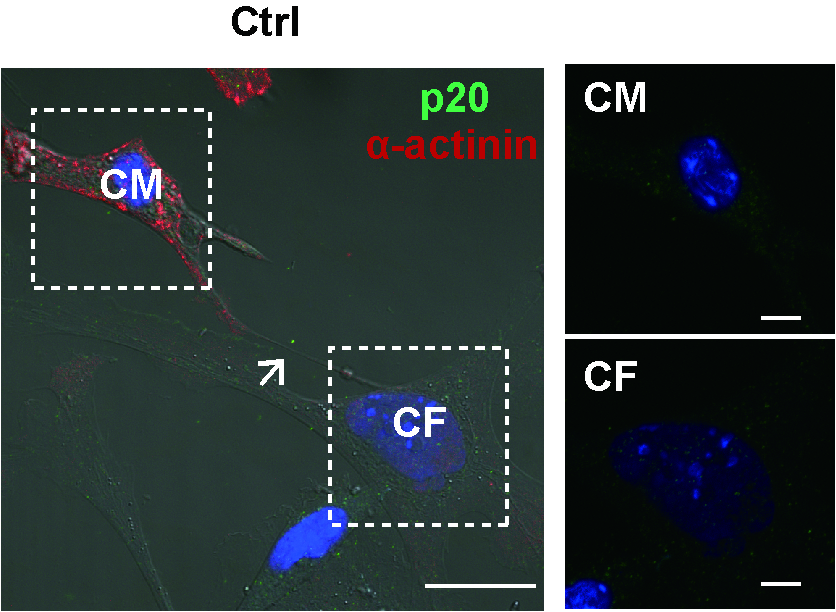

Supplement: Supplementary file 6 — Figure S5 [file 41419_2020_3157_MOESM6_ESM.tif]

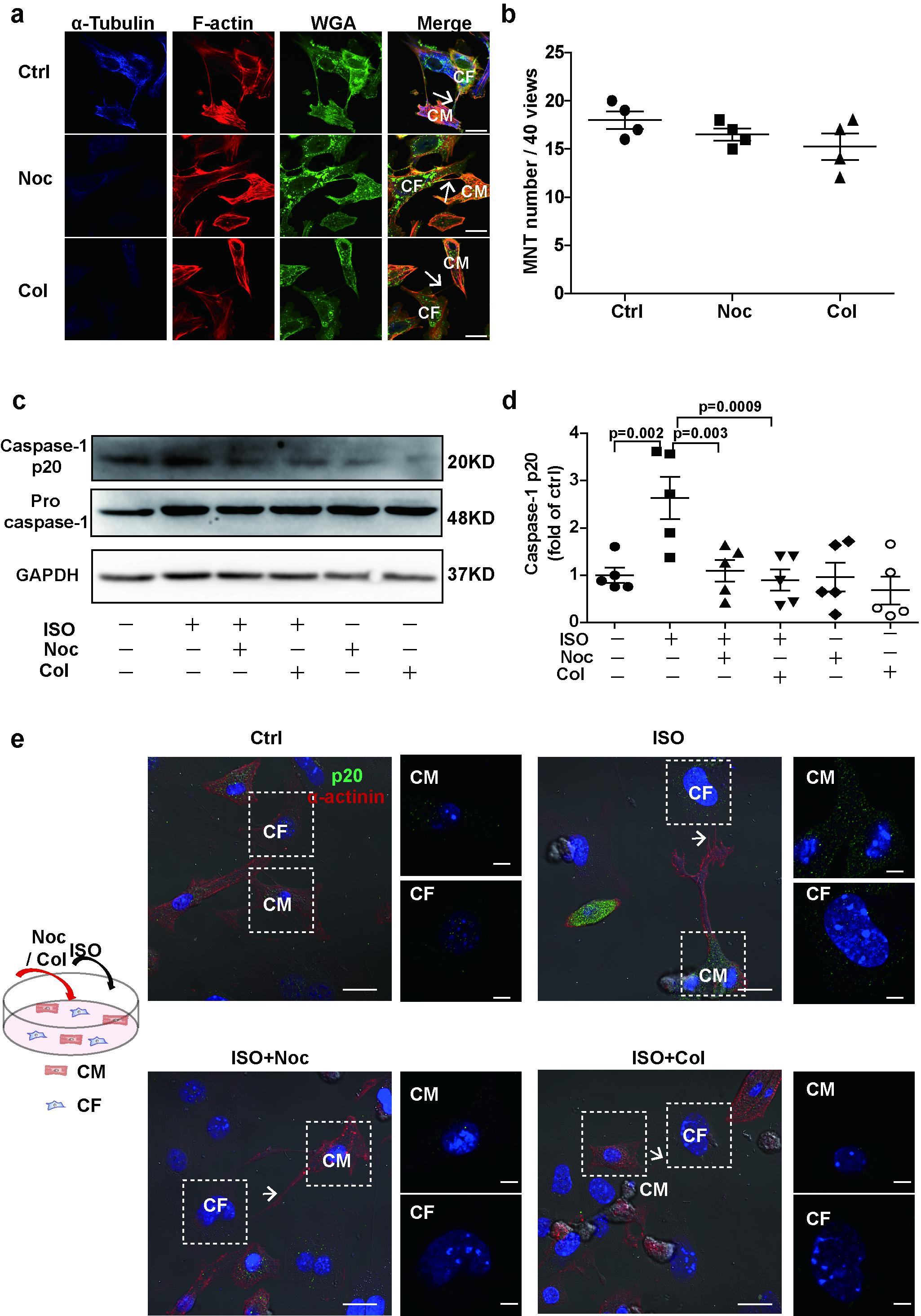

Supplement: Supplementary file 7 — Figure S6 [file 41419_2020_3157_MOESM7_ESM.tif]

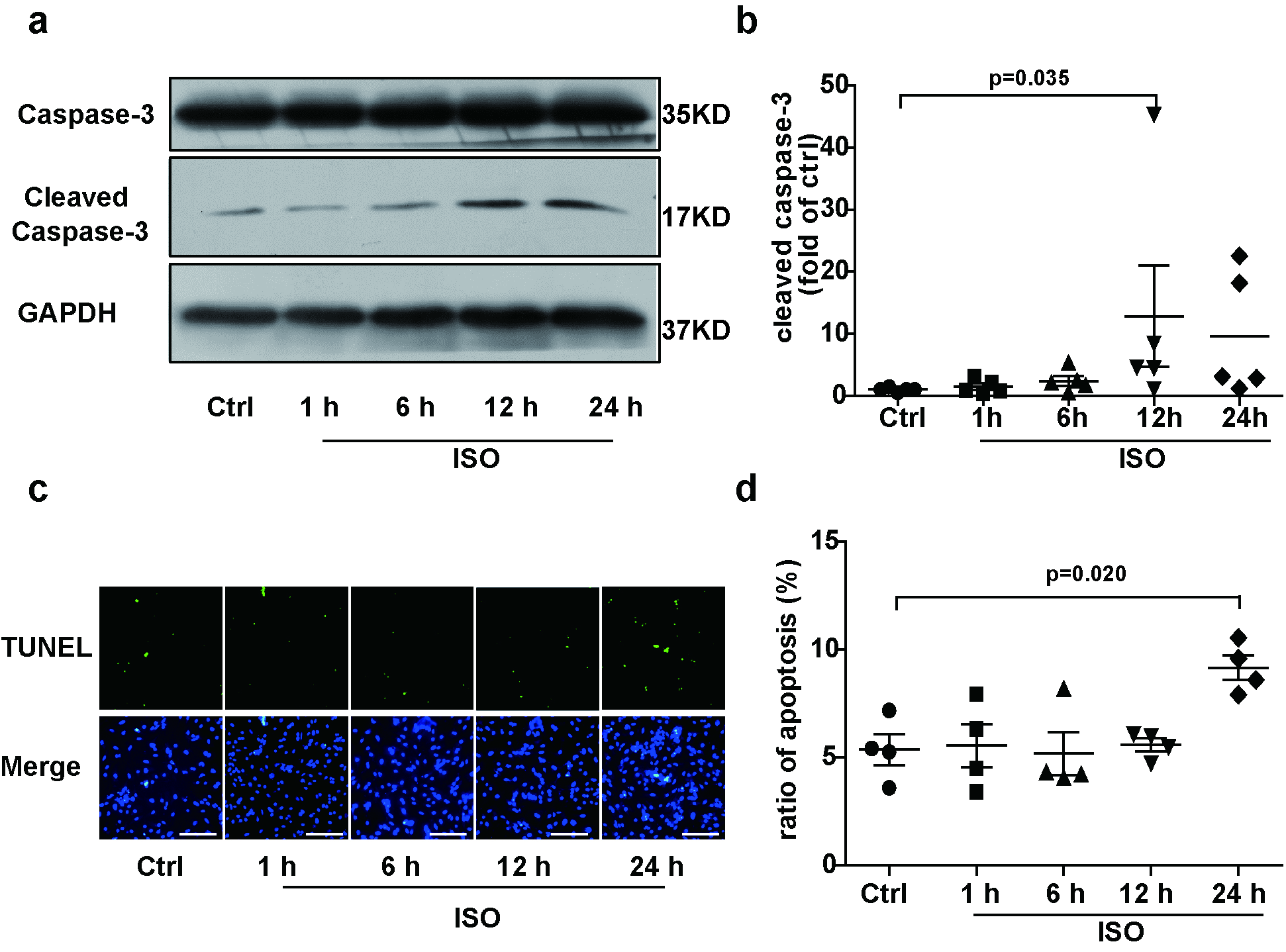

Supplement: Supplementary file 8 — Figure S7 [file 41419_2020_3157_MOESM8_ESM.tif]

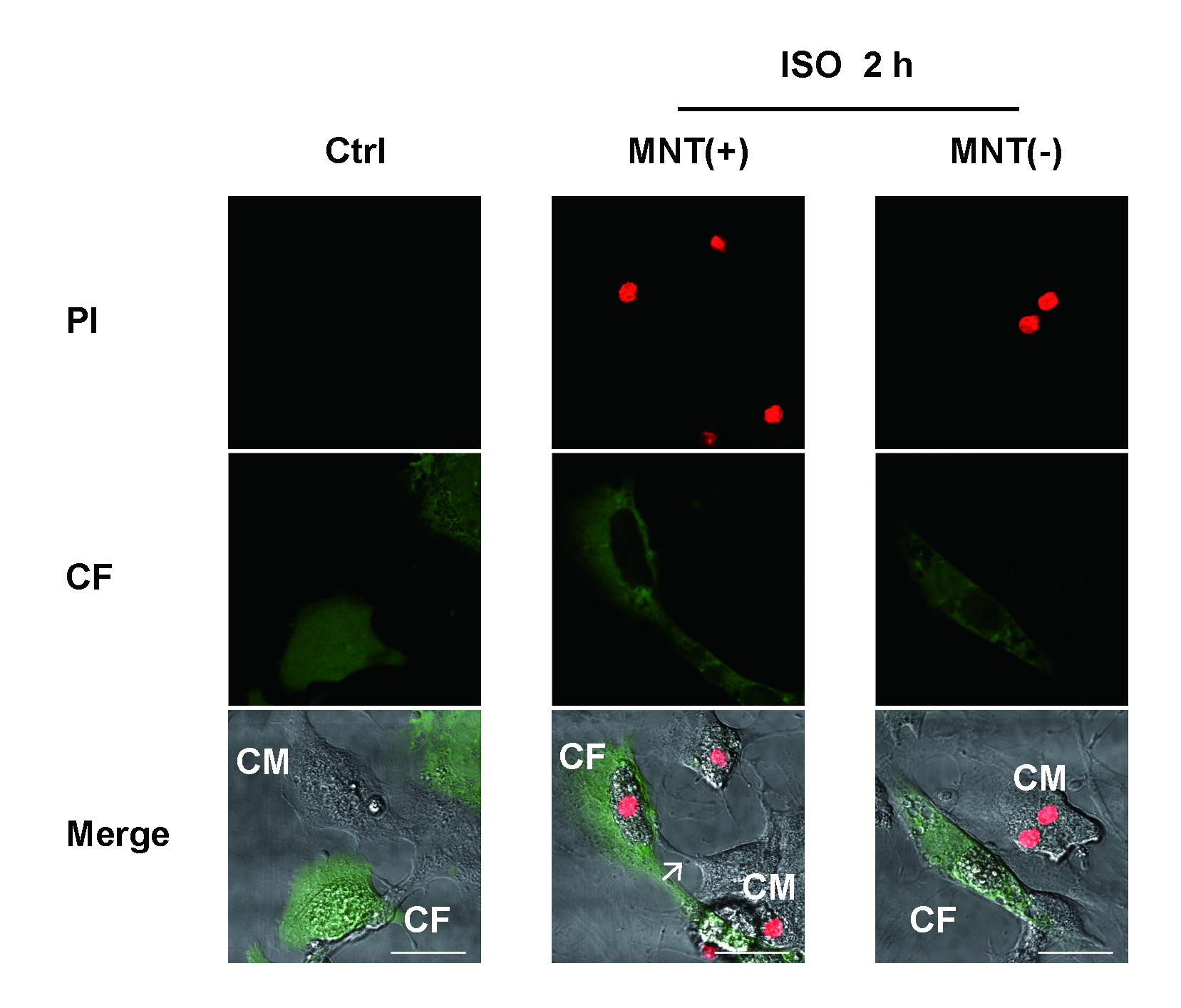

Supplement: Supplementary file 9 — Figure S8 [file 41419_2020_3157_MOESM9_ESM.tif]

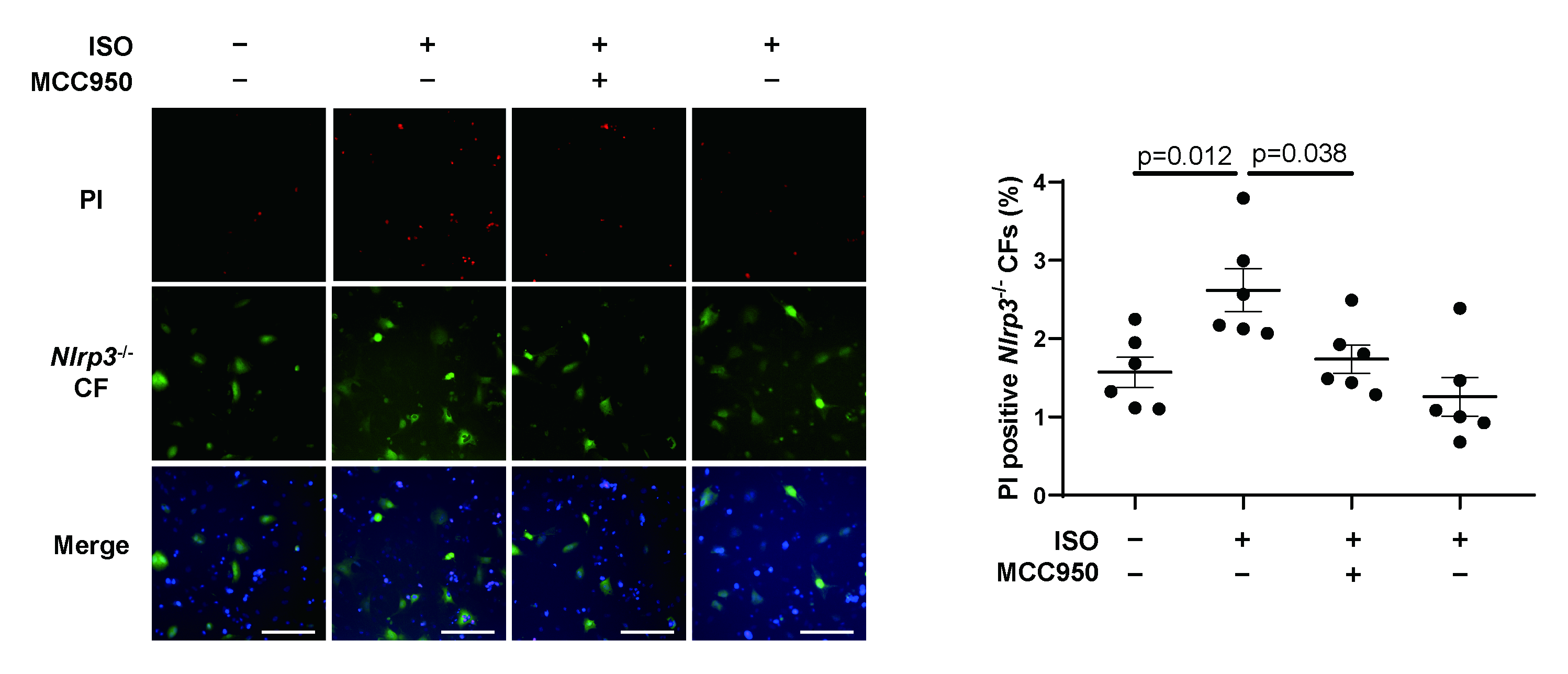

Supplement: Supplementary file 10 — Figure S9 [file 41419_2020_3157_MOESM10_ESM.tif]
